# Detection of Copy Number Variations in Woori-Heukdon Populations with the Illumina PorcineSNP60 Bead-Chip Array

**DOI:** 10.3390/ani15060774

**Published:** 2025-03-09

**Authors:** Yong-Min Kim, Ha-Seung Seong, Seok-Joo Ha, Young-Sin Kim, Jae-Kwon Kim, Heejung Baek, Seona Kwon, Sangwon Yoon, Joon-Hee Lee, Dongwon Seo, Won-Hyong Chung, Joon-Ki Hong, Jung-Woo Choi, Eun-Seok Cho

**Affiliations:** 1 Swine Science Division, National Institute of Animal Science, Rural Development Administration, Cheonan 31000, Republic of Korea; silveraz@korea.kr (Y.-M.K.); kys60m@korea.kr (Y.-S.K.); john8604@korea.kr (J.-K.H.); 2 Animal Breeding and Genetics Division, National Institute of Animal Science, Cheonan 31000, Republic of Korea; seonghs07@korea.kr; 3 Department of Animal Science, College of Animal Life Sciences, Kangwon National University, Chuncheon 24341, Republic of Korea; gk960325@gmail.com (S.-J.H.); wornjs8071@gmail.com (J.-K.K.); hjbaek@tntresearch.co.kr (H.B.); kwonseona23@gmail.com (S.K.); whchung@kangwon.ac.kr (W.-H.C.); 4 Research Institute TNT Research Company, Jeonju 54810, Republic of Korea; swyoon@tntresearch.co.kr (S.Y.); dwseo@tntresearch.co.kr (D.S.); 5 Department of Animal Bioscience, College of Agriculture and Life Sciences, Gyeongsang National University, Jinju 52828, Republic of Korea; sbxjhl@gnu.ac.kr

**Keywords:** copy number variation, Woori-Heukdon, crossbred, Korean duroc, Korean native pig

## Abstract

This study investigated copy number variations (CNVs) in 2112 pigs from five populations: Korean Duroc (DUC), Korean Native Pig (KNP), and their crossbred offspring (F_1_, F_2_, and WRH). CNVs were detected using PennCNV and QuantiSNP, with CNVRuler identifying 698 CNV regions (CNVRs), covering 109 Mb (4.83%) of the porcine genome. Comparison with previous CNV studies on swine revealed CNVR overlap rates ranging from 31.12% (French Yorkshire) to 81.27% (Xiang), and 9.06% newly identified CNVRs. DUC showed the most CNVRs (*n* = 384), followed by WRH (*n* = 225). Meanwhile, F_1_ and F_2_ exhibited far fewer CNVRs (five and seven, respectively). Functional enrichment analysis highlighted various genes overlapping with the CNVRs, including 1236 genes in DUC and 572 genes in WRH, linked to biological processes. The quantitative trait loci (QTLs), overlapping with CNVRs, exhibited particular overlapping with traits such as average daily gain (4.24% of QTLs in DUC, 4.51% of QTLs in WRH). In contrast, KNP, F_1_, and F_2_ populations exhibited a higher frequency of CNVRs containing QTLs overlapped with drip loss. These findings indicate that WRH may inherit growth traits from DUC. This study provides a better understanding of CNVs in the pigs, which can potentially be used in improving genetic merits of pig populations.

## Simple Summary

Copy number variations (CNVs) are segments of DNA that vary in number between individuals or populations. CNVs can potentially influence various traits of interest in livestock species, such as growth, reproduction, and meat quality. Woori–Heukdon (WRH) was developed through a crossbreeding process, mainly using Korean Duroc (DUC) and Korean native pig (KNP). This involved crossing DUC and KNP to produce the F_1_, generation backcrossing DUC with F_1_ to generate F_2_, and subsequently breeding F_2_ with F_1_ to produce WRH. Here, we utilized PennCNV v1.0.5 and QuantiSNP v2.3 software to identify CNVs across the genomes of WRH, its parental breeds (DUC and KNP), and intermediate generations (F_1_ and F_2_). The results revealed distinct CNV patterns across those populations, with WRH CNV regions (CNVRs) showing the highest overlap with quantitative trait loci (QTLs) related to average daily gain (ADG). This suggests that DUC’s rapid-growth traits have been incorporated into WRH through crossbreeding. Additionally, QTLs overlapping with CNVRs in WRH also included some related to meat quality traits, indicating contributions from KNP. These findings highlight the genomic variations associated with the development of WRH and provide valuable insights for breeding programs aimed at enhancing economically important traits in pigs.

## 1. Introduction

Pigs were first domesticated approximately 9000 years ago and have served as an essential source of food worldwide [1,2]. The high demand for pork has contributed to the economic significance of the swine industry. Enhancing pork quality and productivity is essential for the sustainable growth of the pig industry [3]. The Korean native pig (KNP), restored by the National Institute of Animal Science (NIAS) in 1988, is recognized for its superior meat color and high intramuscular fat content [4]. It was subsequently registered as an indigenous pig breed in Korea (DAD-IS, http://www.fao.org/dad-is/) (accessed on 10 December 2023). However, KNP demonstrates lower performance in economically important traits, such as growth rate, limiting its broader utilization in commercial swine production. To address these challenges, the Woori-Heukdon (WRH) was developed through a crossbreeding program involving the Korean Duroc (DUC), which is known for its superior growth rates [5], and KNP. WRH is a synthetic breed combining the genetic strengths of KNP and DUC, resulting in better meat quality and growth rate than KNP. Through three generations of backcrossing, WRH retains 37.5% of the KNP blood composition [5,6]. WRH has shown more improved genetic merits in economic traits, including a 20% faster growth rate and a 25% increase in reproductive traits such as litter size. Additionally, WRH meat exhibits enhanced intramuscular fat content and improved water-holding capacity [7].

This study represents the first comprehensive investigation of CNVs in WRH populations. The investigation of copy number variations (CNVs) is critical for understanding genetic diversity and economically important traits in livestock species. However, research on CNVs in the Woori-Heukdon (WRH) breed, a synthetic pig breed developed in Korea, remains significantly limited. This study aims to address this gap by exploring CNVs across pig populations involved in the development of WRH. CNVs represent structural genomic variations larger than 1 kb, contributing to genetic diversity and influencing complex traits in livestock [8,9]. Recent advancements in genomic technologies now enable the detection of smaller CNVs across the entire genome, providing deeper insights into structural variations [10,11]. CNVs typically encompass larger DNA segments compared to SNPs and insertions and deletions (also known as Indels), and have been shown to affect gene expression, playing a role in genetic diversity, complex traits, and structural variation in genomes [12,13]. CNVs are important for understanding traits of economic importance in livestock, including growth, reproduction, and disease resistance [14,15,16,17,18].

This study aims to investigate CNVs across five pig populations that contributed to the development of WRH, using the Illumina Porcine 60K SNP Bead-Chip array. For reliable detection, two CNV detection programs, PennCNV and QuantiSNP, were employed. These programs analyze CNVs based on Log R Ratio (LRR) and B allele frequency (BAF), interpreted by GenomeStudio [19,20]. We analyzed CNVRs, genes, and quantitative trait loci (QTLs) to understand the genetic structure and identify traits associated with CNVs of each population. Additionally, we explored how WRH’s breeding objectives, like improved growth rate and carcass traits, are reflected in CNV patterns. By analyzing CNVs in Korean pig populations and their relationship to breed-specific traits, this study contributes to the advancing breeding programs and enhancing the quality and productivity of the Korean pig industry.

## 2. Materials and Methods

### 2.1. Samples and Genotyping

This study analyzed genotype data from 2112 pigs, including Korean Duroc (DUC), Korean native pig (KNP), and their crossbred populations: F_1_ (DUC × KNP), F_2_ (F_1_ × DUC), and F_3_ (WRH; F_1_ × F_2_). Genotype data for DUC (N = 1079), KNP (N = 207), F_1_ (N = 14), F_2_ (N = 148), and WRH (F_3_; N = 660) were sourced from a prior study [6]. Additionally, whole blood samples were collected from three individuals, including two DUC (N = 2) and one F_1_ (N = 1), from the National Institute of Animal Science (NIAS), Korea. Genomic DNA was isolated from the samples using the phenol–chloroform extraction method. Genotyping was carried out with the Illumina PorcineSNP60 Bead-Chip (version 2), and SNP data were processed and called using the Illumina GenomeStudio software v2.0 (Illumina, San Diego, CA, USA). The FinalReport and data tables, including the Log R ratio (LRR) and B allele frequency (BAF), were generated by the software and used for the detection of CNVs.

### 2.2. CNV Detection

Multiple programs were used in this study to achieve more accurate CNV detection: PennCNV v1.0.5 (https://penncnv.openbioinformatics.org) (accessed on 8 March 2022) [21] and QuantiSNP v2.3 (accessed on 12 July 2022) [22]. Both tools use Hidden Markov Model (HMM) algorithms to detect CNVs based on Log R ratio (LRR) and B allele frequency (BAF) data from Illumina microarrays [23]. PennCNV employs a transition matrix to model copy number changes between SNP probes, while QuantiSNP uses Bayesian inference to estimate copy number states for each SNP marker and detect CNVs. CNVs were initially filtered independently by each software. For PennCNV, the filtering criteria included LRR values less than 0.3, BAF drift under 0.01, and a GC wave factor below 0.05. CNVs with consecutive SNPs ≥ 3 and CNV length ≥ 10 kb were retained. For QuantiSNP, CNVs were retained if they had included at least 3 consecutive SNPs and a minimum Bayes factor of 10 [24,25,26]. Only autosomal chromosomes were included in the analysis to avoid potential biases from sex chromosomes.

### 2.3. Identification of CNVRs and Functional Analysis

CNVRs were generated using CNVRuler software v1.3.3.2 [27], based on CNVs identified by PennCNV and QuantiSNP. CNVRuler was applied to detect CNVRs from each set of CNVs, optimizing the estimation of CNVR sizes. To minimize false positives, CNVRs that did not meet the density threshold (recurrence parameter < 0.1) were excluded to minimize false positives. The CNVRs identified from each program were subsequently merged using the intersectBed function in BedTools (v2.17.0) [28]. CNVRs identified by PennCNV and QuantiSNP were merged using BedTools with a 1 bp overlap threshold to ensure the inclusion of regions detected by both tools. Overlapping CNVRs were combined into a single region by extending their boundaries to encompass the union of overlapping regions. And CNVRs categorized as gains, losses, or mixed regions based on overlapping results.

Genes overlapping with CNVRs were identified using the NCBI Sus Scrofa11.1 genome assembly (https://www.ncbi.nlm.nih.gov/data-hub/genome/GCF_000003025.6/) (accessed on 12 July 2022). Overlapping genes were defined as those exhibiting any intersection with CNVRs, including overlaps of 1 bp or more. The intersectBed function in BedTools was used to detect these overlaps between CNVRs and genes. Both partial and full overlaps were analyzed to ensure comprehensive coverage of gene–CNVR interactions. Functional annotation clusters (ACs) were generated using the DAVID Bioinformatics Resources tool (https://davidbioinformatics.nih.gov/) (accessed on 21 October 2023) [29]. Genes overlapping with CNVRs were analyzed for enrichment of gene ontology (GO) terms, KEGG pathways, and other functional annotations. DAVID groups related the functional terms into clusters using a clustering algorithm based on shared annotation terms. An enrichment score threshold of 1.3 (corresponding to *p*-value of 0.05) [30] was applied to identify significant clusters. These clusters do not represent direct gene interaction networks, but rather highlight shared biological functions or pathways among the genes analyzed. This allowed the identification of significantly enriched gene ontology (GO) terms and pathways, providing insights into the biological significance of CNVRs in relation to phenotypic traits.

### 2.4. QTL Analysis

The association between CNVRs and QTLs was investigated using data from the pig QTL database (https://www.animalgenome.org/cgi-bin/QTLdb/index) (accessed on 13 July 2023) [31], based on the Sus Scrofa11.1 genome assembly. Overlaps between CNVRs and QTLs were identified with BedTools, providing insights into the potential functional impacts of CNVs on economically important traits.

## 3. Results

### 3.1. Basic Statistics of Copy Number Variations (CNV)

This study employed two CNV detection tools, PennCNV and QuantiSNP, to identify CNVs using the genotypic data of 2112 pigs derived from the Illumina PorcineSNP60 Bead-Chip array. In this study, only autosomal chromosomes were analyzed to investigate CNVs, thereby avoiding potential biases that could arise from the inclusion of sex chromosomes. The CNVs detected by those programs are summarized in Table 1. PennCNV identified a total of 10,375 CNVs, which comprise 3092 gains and 7283 losses, with an average length of 141.9 kb, while QuantiSNP detected 9868 CNVs, including 5429 gains and 4439 losses, with an average length of 422.3 kb. The average length detected with QuantiSNP was significantly greater than that with PennCNV. There were significant differences in CNV counts and the distribution of gains and losses across each of the pig populations, and even between the two detection tools. PennCNV detected a higher proportion of losses than gains (70.2%), whereas QuantiSNP identified slightly more gains (55.0%). In the DUC population, PennCNV and QuantiSNP detected 6905 and 7256 CNVs, respectively, representing the highest CNV counts among the populations analyzed in this study. The average CNV sizes in DUC were 146.1 kb (PennCNV) and 463.6 kb (QuantiSNP). The KNP population had 556 CNVs and 869 CNVs identified by PennCNV and QuantiSNP, respectively. Conversely, the F_1_ crossbred population showed distinctively lower CNV counts than the others, with only 50 CNVs identified by PennCNV and 13 by QuantiSNP. In the WRH population, 2305 CNVs were detected by PennCNV and 1710 by QuantiSNP, showing fewer CNVs than DUC but more than KNP.

### 3.2. Distribution of CNVRs in the Porcine Genome

CNVRs were further retrieved using CNVRuler software based on the CNVs detected by PennCNV and QuantiSNP. From the CNVs identified by PennCNV, a total of 1717 CNVRs were obtained, while QuantiSNP yielded 1127 CNVRs. To enhance the reliability of the findings and reduce potential false positives, regions overlapping between the CNVRs detected by both tools were retrieved using genomic coordinate information obtained from BedTools. This analysis resulted in a total of 681 overlapping CNVRs, classified into three categories: losses (222 CNVRs), gains (52 CNVRs), and mixed regions (407 CNVRs). These overlapping CNVRs covered 4.83% of the porcine autosomes (Table 2).

**Figure 1:**
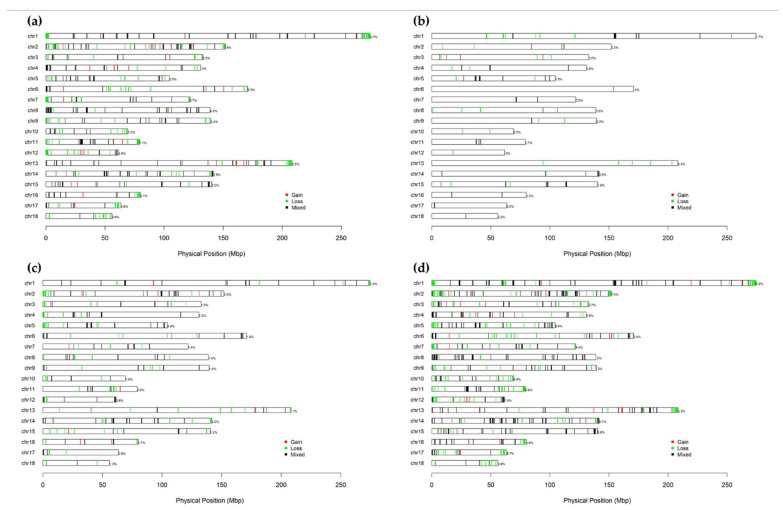
The CNVR maps for pig populations in the 18 autosomes. Three types of CNVR were classified, including gain (red), loss (green), and mixed (black); (**a**) Korean Duroc (DUC); (**b**) Korean native pig (KNP); (**c**) Woori-Heukdon (WRH); (**d**) overall CNVR.

As we expected, the CNVRs were not evenly distributed across the genome; for example, Sus scrofa chromosome (SSC)1 had the highest number of CNVRs (79 CNVRs), covering 5.23% (14.35 Mb) of it. SSC10 had the highest proportion of its genomic region covered by CNVRs, at 10.03% (Figure 1). The DUC population had the highest number of CNVRs, with 384 regions identified, covering 82.01 Mb (3.62%) of the genome. On each of SSC1 and SSC2, 43 CNVRs were detected, while SSC11 showed the highest genomic coverage of CNVRs (7.14%). In addition, in the KNP population, a total of 77 CNVRs were identified, spanning 15.72 Mb (0.69% of the genome); SSC1 had the highest number of CNVRs, with 24 regions identified, while SSC5 showed the greatest coverage (1.80%). Meanwhile, the F_1_ population exhibited a limited number of CNVRs, with only 5 CNVRs (0.03% of the genome), while F_2_ had 7 CNVRs (0.04%). In the F_1_ population, 2 CNVRs were detected on SSC15, the chromosome with the highest CNVR count. For the F_2_ population, 2 CNVRs were identified on both SSC11 and SSC13. Finally, in the WRH population, 225 CNVRs were detected, covering 43.6 Mb (1.92%). In WRH, SSC2 had the highest number of CNVRs, with 24 regions identified, and SSC5 showed the greatest coverage (4.42%) (Figure 1, Table S1).

### 3.3. Functional Annotation and Gene Enrichment Analyses

**Figure 2:**
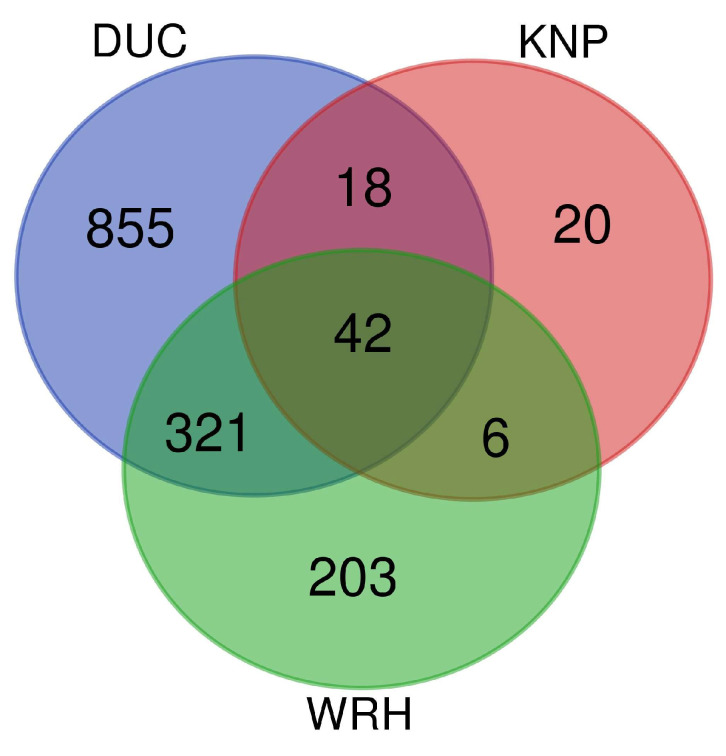
The number of genes located in CNVRs for three breeds: Korean Duroc (Blue), Korean native pig (Red), and Woori-Heukdon (Green).

Genes overlapping with CNVRs were retrieved for each of the populations based on the Sus scrofa 11.1 genome assembly. A total of 1236 genes were retrieved for the DUC population, while 86 genes were identified for KNP and 572 for WRH (Figure 2). In DUC, 323 CNVRs (47.43% of CNVRs) covered the whole or part of gene regions. For KNP and WRH, genes were identified in 59 CNVRs (8.66%) and 187 CNVRs (27.46%), respectively (Table S2). The functional annotation clusters (ACs) represent groups of related functions and pathways among the genes overlapping CNVRs. Although the clusters do not prove direct interactions, it provides insights into shared biological processes in WRH. The analysis revealed nine ACs in DUC, two ACs in KNP, and four ACs in WRH. In the DUC population, several significant ACs were identified: AC1 (enrichment score = 2.91) was related with homeobox genes and transcription factors, while AC3 (enrichment score = 2.45) and AC6 (enrichment score = 1.69) were linked to embryonic skeletal development and neuron differentiation, respectively. Additionally, AC2 (enrichment score = 2.74) was related to the urokinase plasminogen activator receptor and lymphocyte antigen 6. AC4 (enrichment score = 2.04) involved zinc fingers and PHD fingers, indicating roles in molecular interactions and cellular processes. For the KNP population, the two identified ACs were linked to basic protein functions and ubiquitin interactions (AC1: enrichment score = 1.94; AC2: enrichment score = 1.31) (Table S3). The WRH population showed similarities to DUC, with AC1 (enrichment score = 2.12) highlighting zinc finger functions. Additional ACs in WRH were related to cellular communication (AC2: enrichment score = 1.61), the leucine-rich repeat motif (AC3: enrichment score = 1.51), and blood clotting processes (AC4: enrichment score = 1.45) (Table 3).

### 3.4. Overlaps Between CNVRs and QTLs

**Figure 3:**
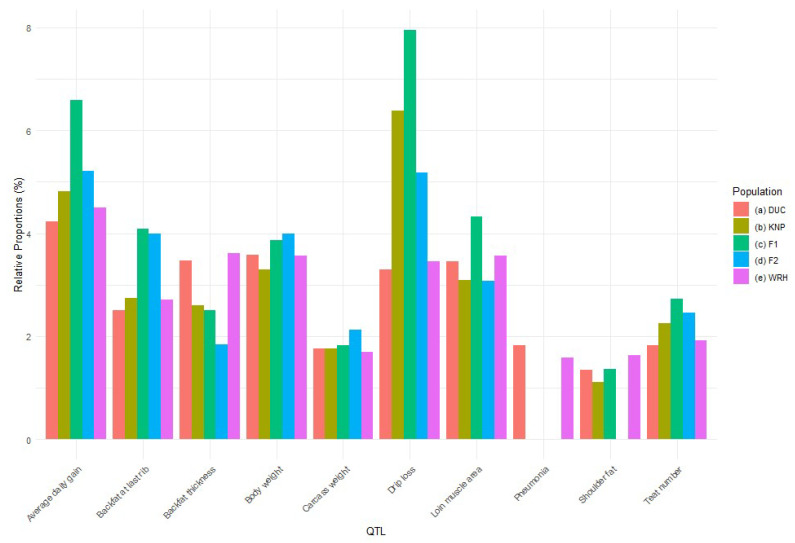
Quantitative trait loci (QTL) number in copy number variant regions (CNVRs); (**a**) DUC; (**b**) KNP; (**c**) F_1_; (**d**) F_2_; (e) WRH.

To explore potential implications of CNVRs for quantitative traits, we used QTL information from the Animal QTL database (https://www.animalgenome.org/cgi-bin/QTLdb/index) (accessed on 13 July 2023). In total, the whole or part of 5292 QTLs overlapped with CNVRs. These QTLs were particularly linked to economic traits in swine, such as average daily gain (4.27% of total QTLs), body weight (3.10% of total QTLs), back fat thickness (3.08% of total QTLs), drip loss (2.83% of total QTLs), and loin muscle area (2.38% of total QTLs). These traits are closely aligned with the primary breeding objectives of WRH, which aim to enhance growth rate and meat quality. In the DUC population, 477 CNVRs overlapped with QTLs, which were particularly associated with traits like average daily gain (4.24% of total QTLs) and body weight (3.58% of total QTLs). Meanwhile, in the KNP population, 107 CNVRs overlapped with QTLs, most commonly associated with the trait drip loss (6.39%). In each of the F_1_ and F_2_ populations, the QTLs showed overlaps with six CNVRs and were, again, primarily linked to drip loss. Finally, in the WRH population, 302 CNVRs overlapped with QTLs, with the highest proportion of QTLs being linked to average daily gain (4.51%), followed by average back fat thickness (3.61%) (Figure 3).

### 3.5. Comparison with CNVRs Identified in Previous Studies

When comparing our identified CNVRs with those reported in previous studies on pigs, we observed 577 CNVRs (90.94%) overlapping with previously published studies [32,33,34,35,36] (Table 4). The overlap rates across studies ranged from 31.12% to 81.27%. The longest overlapping CNVRs (81.27%) were observed in comparison with Liu et al. (2024) [36]. The second-highest overlap was found with Qiu et al. (2021) [34], showing a 56.46% overlap. Lin et al. (2024) [35] had the smallest overlap, with an overlap rate of 31.12%. Additionally, 9.06% of the 104 CNVRs (9.91 Mb) identified in our study were newly discovered. Overlapping CNVR events were defined using the criterion that two CNVRs must share at least one base pair.

## 4. Discussion

In this study, we initially detected 10,375 CNVs (copy number variations) by PennCNV and 9868 by QuantiSNP across five pig populations (Table 1). By analyzing the clustering of these CNVs, we retrieved a total of 681 CNVRs (CNVR regions), covering a cumulative span of 109.39 Mb, which represents 4.83% of the Sus scrofa reference genome assembly (Table 2). Of the total CNVRs, the proportion of losses of copy numbers was significantly higher than the proportion of gains across all five populations, with losses making up 35.4% of total CNVRs. Such a tendency as in this observation coincides with previous studies, as both programs utilize Log R ratio (LRR) and B allele frequency (BAF) values to identify CNVs. LRR is the log2 ratio of observed signal intensity to the expected intensity for a given SNP, and is commonly used to infer copy number variations. BAF is a metric derived from SNP arrays that measures the proportion of signal intensity attributed to each allele and helps detect genomic imbalances [23]. Previous studies suggested that deletions are detected more frequently than duplications for technical reasons. Specifically, single nucleotide polymorphism (SNPs) deletions tend to create greater proportional changes in the LRR values, making them easier to detect than duplications [37,38]. Here, gains accounted for 8.9% of total CNVRs, and the remaining 55.7% were mixed regions with both gains and losses. These results coincide with previous studies that used the PorcineSNP60 Bead-Chip array, which similarly found more losses than gains [39,40,41].

In this study, a comparison of CNVRs identified in WRH and its parental breeds with previous studies revealed that approximately 9.91 Mb (9.06%) of the total 109.39 Mb CNVRs were unique to this research. In the unique region, 7 CNVRs were common across DUC, KNP, and WRH, while 50 CNVRs were unique to DUC, 5 were unique to KNP, and 29 were unique to WRH (Table S4). When comparing the results of this study with previous studies, the highest overlap rate was observed with Liu et al. (2024) [36] using Xiang pigs, with an overlap of approximately 81.27%. Conversely, the lowest overlap rate (31.12%) was observed with Lin et al. (2024) [35] in the French Yorkshire pig. The higher overlap with the Xiang breed can be attributed to the broader CNVRs identified in the study by Liu et al. (2024) [36], leading to more shared regions. However, despite detecting a greater number of CNVRs, the study by Lin et al. (2024) [35] using French Yorkshire pigs showed the least overlap with our findings compared to both Dong et al. (2015) [32] and Wang et al. (2020) [33]. This discrepancy may reflect breed-specific CNVR patterns or methodological differences across studies (Table 4).

We observed some distinct patterns in the CNVR counts, as well as CNVR lengths, among the populations. In the de facto founder population (parental generation) from which WRH was established, DUC exhibited the highest number of CNVRs (384 CNVRs, covering 3.62% of the genome), while KNP had significantly fewer CNVRs (77 CNVRs, 0.69%). Meanwhile, the WRH population showed an intermediate number between them (225 CNVRs, 1.92%), suggesting that its genomic pattern was influenced by both parental breeds (DUC and KNP). The F_1_ and F_2_ crossbred populations displayed the fewest CNVRs (F_1_: five CNVRs, 0.03%; F_2_: seven CNVRs, 0.04%), which may be attributable, at least in part, to their small sample sizes here. Through the process of crossbreeding between DUC and KNP, genetic recombination and selection pressures altered the frequency and distribution of specific CNVs, leading to the spread of CNVs associated with growth and meat quality throughout the WRH population. Furthermore, with each generation, reductions in genetic diversity and the fixation of specific gene clusters occurred, resulting in the WRH population exhibiting intermediate, yet unique, genomic characteristics. To further explain the functional implications of the identified CNVRs, we analyzed the distribution of genic CNVRs. Overall, 438 CNVRs (80.76 Mb) overlapped with annotated genes, indicating their potential functional consequences. We also quantified the proportion of CNVRs that overlapped with quantitative trait loci (QTLs) are genomic regions associated with economically significant traits in pigs. CNVRs identified in WRH exhibited the highest overlap with average daily gain (ADG) of QTLs (670 QTLs, 4.51%), reflecting the influence of growth-related traits inherited from DUC, while the KNP population showed a higher proportion of overlaps with drip loss-related QTLs (275 QTLs, 6.39%), underscoring their contribution to meat quality traits in WRH (Table S2 and Figure 3). To statistically evaluate the observed overlaps between CNVRs and QTLs, a chi-squared test was conducted. The analysis revealed a highly significant enrichment (*p* < 2.2 × 10^−16^) of QTLs within CNVRs. Specific CNVRs were identified in each population, unique to their genetic background. DUC had 319 CNVRs exclusively found in this population, KNP had 30, and WRH had 148. Even in populations with fewer CNVRs, like F_1_ and F_2_, some specific CNVRs were identified. For instance, F_1_ had CNVR_590 (SSC15: 12,021,045–12,094,123), while F_2_ had three specific CNVRs: CNVR_444 (SSC11: 41,414,301–41,476,323), CNVR_507 (SSC13: 148,928,467–149,139,352), and CNVR_525 (SSC13: 203,329,171–203,372,139). Their limited sample sizes might influence the smaller numbers of CNVRs identified in F_1_ and F_2_. Indeed, previous studies on pig CNVs detected using PennCNV demonstrated that smaller sample sizes tend to result in fewer detected CNVs [42,43].

To assess the impact of sample size on CNV detection, we conducted analyses limiting the sample size to 14 individuals per population. Under these conditions, both PennCNV and CNVRuler detected fewer CNVRs across all populations (Table 5). In fact, KNP exhibited fewer CNVRs than F_1_ and F_2_, while DUC and WRH consistently showed higher CNVR counts. This pattern underscores the importance of sample size in CNV detection, highlighting the need for a sufficiently large sample size to ensure accurate results. WRH exhibited more CNVRs than F_1_ and F_2_, and the CNVR count in the offspring groups (F_1_, F_2_, and WRH) was higher than that of KNP, but lower than that of DUC. This suggests that DUC, which shows a higher number of CNVRs, has a greater influence on CNVR patterns as crossbreeding progresses (Table 5). Relatively few of the CNVRs overlapped among all five of the DUC, KNP, F_1_, F_2_, and WRH populations (Table S2). This may be attributable to the smaller sample sizes in the F_1_ and F_2_ populations, which could have reduced the statistical power for detecting CNVs and identifying overlaps with other populations (Table 2 and Table 5).

The chromosomal distribution of CNVRs revealed that the highest CNVR coverage in DUC was on SSC11 (7.14%), while for KNP and WRH, it was on SSC5 (1.80% and 4.42%, respectively). Analysis of the distribution of CNVRs on SSC5 that were shared between KNP and WRH identified genes related to olfactory function. These CNVRs also overlapped with QTLs linked to traits such as meat color, back fat thickness, and loin muscle area. This suggests that WRH may inherit some of KNP’s beneficial meat quality traits, although this contribution is relatively minor, accounting for less than 1% of the total QTLs.

Functional annotation and gene enrichment analyses revealed that WRH exhibited four functional clusters related to CNVRs, including zinc finger genes (AC1: enrichment score = 2.12), similar to DUC’s AC4. Notably, 59.02% of genes in WRH’s CNVRs overlapped with DUC, indicating a genetic influence from DUC. A shared CNVR between DUC and WRH, CNVR_181 (SSC4: 274,403–1,232,672), contained the diacylglycerol acyltransferase-1 (DGAT1) gene, which is known to influence meat quality by increasing intramuscular fat content [44,45]. Additionally, AC4 was linked to von Willebrand factor (VWF), a protein crucial for blood clotting [46,47]. This finding suggests that DUC has contributed to blood clotting traits in WRH.

QTLs overlapping with CNVRs were linked to 494 traits, particularly average daily gain, back fat thickness, body weight, and drip loss. Average daily gain QTLs were particularly prevalent in both DUC (4.24%) and WRH (4.51%) populations, indicating that WRH may have inherited growth-related traits from DUC. Additionally, DUC and WRH shared 140 CNVRs containing QTLs. Drip loss, an indicator of meat quality closely associated with water-holding capacity and texture [48], was the trait showing the highest frequency of overlapping QTLs in the KNP population. Some CNVRs containing drip loss QTLs were shared between KNP and WRH. It is known that the trait of drip loss has high heritability [49]. This suggests that KNP may have contributed to the meat quality traits in WRH. These results align with the main objectives of breeding WRH, namely, to combine the rapid growth traits of DUC with the superior meat quality traits of KNP.

The average daily gain QTLs in WRH is consistent with previous reports highlighting WRH’s superior growth rate compared to KNP, achieving a 20% faster growth rate due to its DUC lineage. Similarly, the identification of QTLs associated with drip loss in KNP and WRH aligns with prior findings that emphasize KNP’s contribution to meat quality traits, such as enhanced intramuscular fat content and water-holding capacity [7].

These findings offer valuable insights for breeding programs aiming to improve specific traits through selective crossbreeding. The success of this breeding strategy is evidenced by WRH exhibiting improved growth performance while maintaining favorable meat quality traits. Also, 9.06% of CNVRs were unique to this study, offering potential targets for future breeding efforts. These results provide a solid foundation for leveraging CNVs in improving growth performance and meat quality in pig populations.

## 5. Conclusions

In this study, we conducted a comprehensive CNV analysis on the WRH population, analyzing CNVs across 2112 pigs, including DUC, KNP, and their crossbred populations (F_1_, F_2_, and WRH). The results demonstrate that CNVs exhibit distinct patterns across the populations studied, with WRH showing a mixture of genetic influences from both parental breeds. The greatest number of CNVRs in DUC and the overlap of these CNVRs with those in WRH suggest that DUC may have had a substantial influence on the genomic structure of WRH, particularly in growth-related traits, such as average daily gain. The genomic contribution of KNP was less pronounced than that of DUC. However, we found evidence that some genomic characteristics of KNP have been inherited by WRH, For instance, WRH and KNP share 31 CNVRs (4.28 Mb) that are potentially associated with meat quality traits, such as drip loss. These CNVs are likely to contribute to economically significant traits such as growth, carcass quality, and meat characteristics, aligning with the breeding goals for WRH. Additionally, this research offers important genetic insights into pig populations and is likely to serve as a key resource for future genomic studies in swine breeding programs. To the best of our knowledge, this study represents the first CNV analysis conducted on the WRH population.

## Figures and Tables

**Table 1:** Basic statistics of copy number variations detected by PennCNV and QuantiSNP.

Population	N	Tool	CNV Number	Gain	Loss	Average Size (bp)
DUC	1020	PennCNV	6905	2218	4687	146,131
	1018	QuantiSNP	7256	4598	2658	463,610
KNP	172	PennCNV	556	147	409	108,009
	177	QuantiSNP	869	227	642	276,279
F_1_	14	PennCNV	50	23	27	96,351
	8	QuantiSNP	13	7	6	152,315
F_2_	135	PennCNV	559	201	358	126,939
	13	QuantiSNP	20	3	17	95,532
WRH	484	PennCNV	2305	503	1802	142,742
	453	QuantiSNP	1710	594	1116	399,593
Overall	1825	PennCNV	10,375	3092	7283	141,867
	1669	QuantiSNP	9868	5429	4439	442,257

**Table 2:** Basic statistics of CNV regions (CNVRs) of five pig breeds.

Population	CNVR	Loss	Gain	Mixed	Average (bp) ^1^	Total Length (Mb)	Percent (%) ^2^
DUC	384	129	38	217	213,569	82.01	3.62
KNP	77	31	7	39	204,205	15.72	0.69
F_1_	5	3	2	0	128,545	0.64	0.03
F_2_	7	2	0	5	127,441	0.89	0.04
WRH	225	82	15	128	193,781	43.6	1.92
Overlap	681	222	52	407	160,629	109.39	4.83

^1^ Average length of CNVRs. ^2^ CNVR frequency in pig genome (total length in CNVR/pig genome length).

**Table 3:** Gene annotation and enrichment scores for WRH.

Annotation Cluster	Enrichment Score	Category	Term	*p*-Value	Genes	Fold Enrichment ^1^
Annotation Cluster 1	2.120	UP_SEQ_FEATURE	DOMAIN:PHD-type	0.001	RAI1, KDM4A, BRD1, DPF2, CXXC1, JADE1, PHRF1, TRIM66, PHF21B	4.572
		SMART	SM00249:PHD	0.001	RAI1, KDM4A, BRD1, DPF2, CXXC1, JADE1, PHRF1, TRIM66, PHF21B	4.400
		INTERPRO	IPR001965:Zinc finger, PHD-type	0.001	RAI1, KDM4A, BRD1, DPF2, CXXC1, JADE1, PHRF1, TRIM66, PHF21B	4.324
		INTERPRO	IPR019787:Zinc finger, PHD-finger	0.009	BRD1, DPF2, CXXC1, JADE1, PHRF1, TRIM66, PHF21B	3.938
		INTERPRO	IPR011011:Zinc finger, FYVE/PHD-type	0.013	KDM4A, BRD1, HGS, DPF2, CXXC1, JADE1, PHRF1, TRIM66, PHF21B	2.872
		INTERPRO	IPR013083:Zinc finger, RING/FYVE/PHD-type	0.079	KDM4A, BRD1, RNF25, CXXC1, JADE1, LOC110255327, PHRF1, PHF21B, ANAPC11, RAI1, HGS, TRIM3, DPF2, RNF166, TRIM66, TRIM67, RNF220	1.560
		INTERPRO	IPR019786:Zinc finger, PHD-type, conserved site	0.188	BRD1, CXXC1, JADE1, TRIM66	2.672
Annotation Cluster 2	1.610	GOTERM_BP_DIRECT	GO:0071404~cellular response to low-density lipoprotein particle stimulus	0.002	SYK, CD81, ITGB2, SOCS5	14.231
		GOTERM_BP_DIRECT	GO: 0031623~receptor internalization	0.040	SYK, CD81, ITGB2, RAMP1	5.218
		GOTERM_MF_DIRECT	GO:0005178~integrin binding	0.159	CD151, SYK, CD81, ITGB2, P4HB	2.375
Annotation Cluster 3	1.509	UP_KW_DOMAIN	KW-0433~Leucine-rich repeat	0.011	RNH1, LRRC24, FLII, LRRC27, FBXL17, SCRIB, ELFN1, PIDD1, NLRP6, LRRTM3, LGR4, DRC3, FBXL6	2.316
		INTERPRO	IPR001611:Leucine-rich repeat	0.014	LRRC14, LRRC56, RNH1, LRRC24, FLII, LRRC27, FBXL17, TONSL, SCRIB, ELFN1, PIDD1, LRRTM3, LGR4, DRC3	2.161
		SMART	SM00369:LRR_TYP	0.076	PIDD1, LRRC24, LRRTM3, LRRC27, FLII, SCRIB, ELFN1, DRC3, LGR4	2.029
		INTERPRO	IPR003591:Leucine-rich repeat, typical subtype	0.083	PIDD1, LRRC24, LRRTM3, LRRC27, FLII, SCRIB, ELFN1, DRC3, LGR4	1.994
Annotation Cluster 4	1.451	UP_SEQ_FEATURE	DOMAIN:VWFD	0.003	MUC2, SUSD2, MUC5AC, MUC6	8.164
		SMART	SM00216:VWD	0.006	MUC2, SUSD2, MUC5AC	10.237
		INTERPRO	IPR025155:WxxW domain	0.008	MUC2, MUC5AC	21.379
		INTERPRO	IPR001846:von Willebrand factor, type D domain	0.008	MUC2, SUSD2, MUC5AC	9.502
		INTERPRO	IPR002919:Trypsin inhibitor-like, cysteine-rich domain	0.026	MUC2, MUC5AC	11.661
		SMART	SM00832:SM00832	0.034	MUC2, MUC5AC	10.040
		INTERPRO	IPR014853:Uncharacterised domain, cysteine-rich	0.041	MUC2, MUC5AC	9.163
		SMART	SM00215:VWC_out	0.051	MUC2, MUC5AC	8.157
		SMART	SM00041:CT	0.089	MUC2, MUC5AC	5.933
		INTERPRO	IPR006207:Cystine knot, C-terminal	0.107	MUC2, MUC5AC	5.345
		UP_KW_LIGAND	KW-0186~Copper	0.137	MUC2, DBH, MUC5AC	3.080
		SMART	SM00214:VWC	0.207	MUC2, MUC5AC	3.528
		INTERPRO	IPR001007: von Willebrand factor, type C	0.231	MUC2, MUC5AC	3.289

^1^ Fold enrichment represents the ratio of observed genes in the input list to the expected number of genes in the respective functional category. The expected number is calculated based on the reference genome distribution.

**Table 4:** Comparison between CNVRs detected in the study with those in the previous reports.

Study	Platform	CNV Analysis Algorithm	Reference Genome	Sample	Breed Number	CNVR	Total (Mb)	Overlapped CNVR Count with the Present Study	Overlapped CNVR Percentage with the Present Study
Dong, K. et al. (2015) [32]	Porcine SNP 60K Bead-Chip	PennCNV	Sscrofa 10.2	96	Tibetan Dahe Wuzhishan	105	16.71	41.84	38.24%
Wang et al. (2020) [33]	Porcine SNP 80K Bead-Chip	PennCNV	Sscrofa 10.2	1199	Large White	312	57.76	47.74	43.64%
Qiu et al. (2021) [34]	GeneSeek Porcine 50 K SNP chip	PennCNV	Sscrofa 11.1	9484	Duroc (U.S and Canadian)	953	246.89	61.76	56.46%
Lin et al. (2024) [35]	GeneSeek Porcine 80K SNP chip	PennCNV	Sscrofa 11.1	659	French Yorkshire	429	66.78	34.04	31.12%
Liu et al. (2024) [36]	Illumina HiSeq2500 platform	CNVnator CNVcaller	Sscrofa 11.1	83	Xiang	7893	1157.34	88.90	81.27%
Present study	Porcine SNP 60K Bead-Chip	PennCNVQuantiSNP	Sscrofa 11.1	2112	Duroc KNP F_1_ F_2_ WRH	681	109.39	-	-

**Table 5:** Analysis of CNVRs in pig populations with 14 individuals per breed.

Breed		CNVR	Loss	Gain	Mixed	Average	Total Length (Mb)	Percentage (%)
F_1_		23	11	12	0	983,320.30	2.26	0.11
DUC	First	52	32	18	2	121,326.13	6.31	0.28
	Second	92	80	11	1	170,862.71	15.72	0.69
	Third	115	99	16	0	194,439.77	22.36	0.99
KNP	First	12	5	6	0	60,495.55	0.67	0.03
	Second	18	8	10	0	96,363.06	1.73	0.08
	Third	21	12	8	1	93,529.81	1.96	0.09
F_2_	First	21	7	14	0	105,154.24	2.21	0.1
	Second	24	15	9	0	89,116.71	2.14	0.09
	Third	27	14	12	1	95,181.78	2.57	0.11
WRH	First	17	11	6	0	108,536.53	1.85	0.08
	Second	53	50	3	0	204,841.42	10.86	0.48
	Third	99	87	11	1	284,207.04	28.14	1.24

## Data Availability

The datasets of the current study are available from the corresponding author upon reasonable request and with permission of National Institute of Animal Science, RDA, in the Republic of Korea.

## Supplementary Materials

The following supporting information can be downloaded at: https://www.mdpi.com/article/10.3390/ani15060774/s1, Table S1: Distribution of copy number variation region (CNVR) in pigs (Sus Scrofa) autosomes; Table S2: CNVRs Detailed CNVR Data: Gene and QTL Distribution Across Five Populations in five pig populations; Table S3: Annotation Clusters and Enrichment Scores from DAVID Analysis; Table S4. Comparison of CNVRs Identified in This Study with Previously Reported Pig CNVRs.
